# Hereditary Spherocytosis as an Atypical Presentation of Anemia in Ulcerative Colitis

**DOI:** 10.1097/PG9.0000000000000086

**Published:** 2021-06-15

**Authors:** Moo Cho, Suzanne Tucker, Lillian Choi

**Affiliations:** From the * Department of Gastroenterology, Hepatology and Nutrition, University of California, San Diego, CA; † Department of Pathology, University of California, San Diego, CA.

**Keywords:** ulcerative colitis, anemia, hemosiderosis

## Abstract

Anemia is encountered in up to two-thirds of all patients with inflammatory bowel disease (IBD). We are reporting a case of a 9-year-old female with history of very early onset IBD ulcerative colitis, and primary sclerosing cholangitis who was found to have hereditary spherocytosis as the etiology of her anemia. Despite good clinical response to IBD therapy, she continued to have persistent normocytic anemia. Liver biopsy and magnetic resonance cholangiopancreatography for uptrending liver transaminases demonstrated iron deposition which led to a T2-weighted magnetic resonance imaging study that quantified significant iron deposition in her liver and kidneys. Without any history of blood transfusions, these findings were concerning for hereditary hemochromatosis, but the hereditary hemochromatosis gene test was negative. Whole genome sequencing identified a pathogenic de novo variant consistent with hereditary spherocytosis.

**Table of Contents Summary:**

A novel presentation of anemia in inflammatory bowel disease

## INTRODUCTION

Anemia is a common manifestation of inflammatory bowel disease, occurring in up to two-thirds of patients.^1^ The most common causes include iron deficiency anemia and anemia of chronic disease. Factors that tend to contribute to iron deficiency anemia include chronic gastrointestinal bleeding and poor absorption of iron.^2^

## CASE REPORT

**FIGURE 1.**
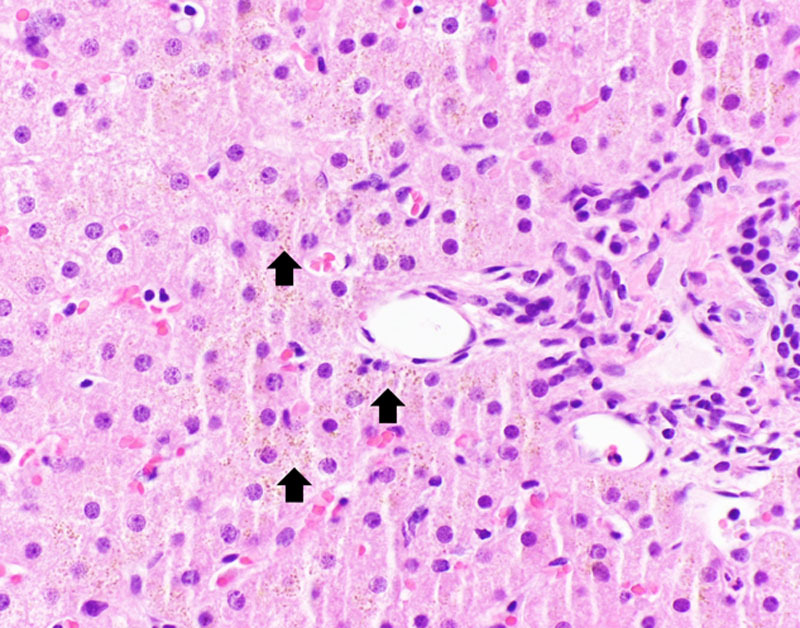
H&E stain section. Small brown granules (arrow) demonstrate iron deposition and are in a periportal distribution within the hepatocytes rather than in Kupffer cells.

**FIGURE 2.**
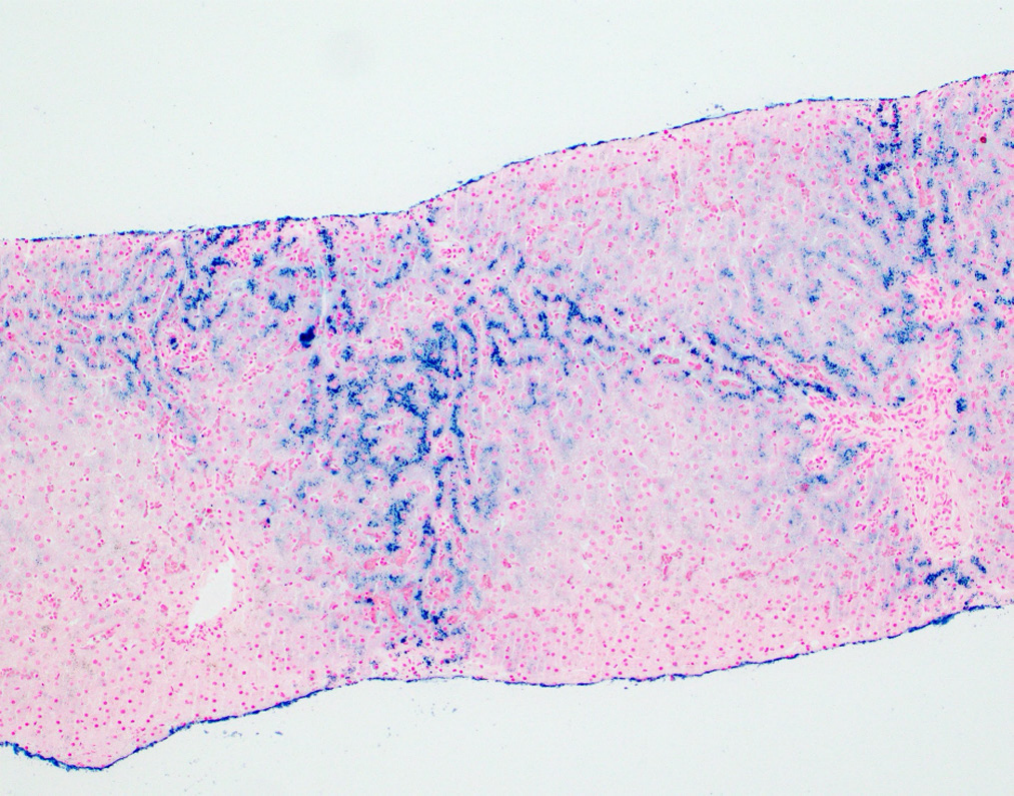
Prussian Blue iron stain of liver. The pericannicular or “chicken wire” distribution of Prussian blue positive granules in periportal hepatocytes (zone 1 distribution) is suggestive of hereditary hemochromatosis.

**FIGURE 3.**
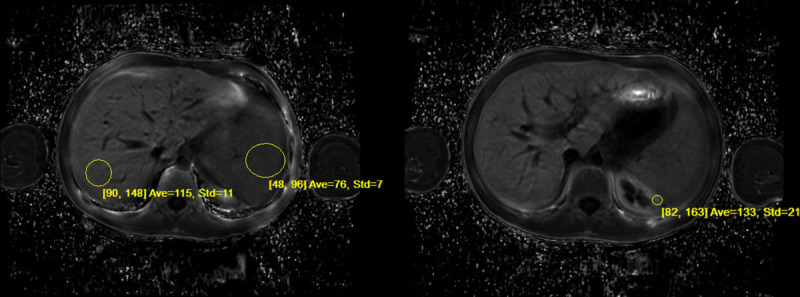
Ideal IQ R2* Sequence: MRI measurements estimating hepatic iron concentrations in the liver, spleen, and kidney using modified gradient echo sequences with the GE Discovery 450. Circled areas on image show areas used for calculating iron concentrations: Liver 3.1 mg per gram of liver tissue, spleen 2.4 mg per gram of spleen tissue, and renal 3.0 mg per gram of kidney tissue.

We report a 9-year-old female with very early onset inflammatory bowel disease (VEO sIBD) and primary sclerosing cholangitis (PSC) with persistent normocytic anemia. Her treatment consisted of 6-mercaptopurine, rifaximin, and sulfasalazine for her IBD and ursodiol for her PSC. She had clinically done well on this therapy with consistently normal erythrocyte sedimentation rate and C-reactive protein; however, she had a persistent normocytic anemia despite no hematochezia or melena. Her liver enzymes and bilirubin were uptrending, but her physical examination was normal with no jaundice, scleral icterus, or hepatosplenomegaly. Given her PSC diagnosis, she underwent a magnetic resonance cholangiopancreatography (MRCP) and liver biopsy. Liver biopsy showed increased iron deposition within the hepatocytes in a pattern suggestive of hereditary hemochromatosis (Figs. 1 and 2). Quantitative iron determination in the liver biopsy revealed elevated iron level of 1654 µg/g and an iron-index of 3.4 µmol/g/year (normal range for iron-index is <1.0 µmol/g/year). T2-weighted magnetic resonance imaging (MRI) demonstrated significant iron deposition above normal range in the liver, spleen, and kidneys (Fig. 3). MRI of the head and heart were normal, and the hereditary hemochromatosis gene test was negative. She had no history of blood transfusions, and therefore, further evaluation was pursued for additional etiologies of iron overload.

The patient had an elevated reticulocyte count, low haptoglobin level, and elevated indirect bilirubin level, suggestive of a hemolytic process. Direct coombs test was negative. Peripheral smear demonstrated polychromasia, without evidence of hemolysis. Evaluation for porphyria cutanea tarda was negative with elevated plasma porphyrins but normal urine and fecal porphyrins. Whole genome sequencing identified a pathogenic de novo variant in the SPTB gene, which encodes a spectrin protein consistent with hereditary spherocytosis.

## DISCUSSION

This is the first reported case of hereditary spherocytosis as the cause of chronic anemia in a patient with IBD. Although anemia is a common finding, the etiology can vary widely. Iron deficiency anemia is the most common cause of anemia in IBD.^2^ Factors contributing to iron deficiency anemia include iron malabsorption, malnutrition, and gastrointestinal bleed. It is also important to note that medications used to treat IBD, in particular, the 2 medications our patient is taking, 6-MP and sulfasalazine, can contribute to anemia. Azathioprine and 6-MP induce myelosuppression, and sulfasalazine can decrease folate absorption. Anemia of chronic disease often develops in patients without an identifiable etiology.

Our patient had chronic anemia and incidental finding of iron overload during routine follow-up of the patient’s PSC. The liver biopsy demonstrated iron deposition in the periportal hepatocytes rather than in the Kupffer cells. This finding is consistent with hemochromatosis.^3^ The T2-weighted MRI demonstrated an iron index greater than 1.9 which further supported the diagnosis of hemochromatosis.^4^ However, the hereditary hemochromatosis gene test was negative (sensitivity >90%, specificity >98%, Quest Diagnostics Nichols Institute). The T2 weighted MRI was also notable for renal hemosiderosis, typically a marker of chronic intravascular hemolysis, and has been reported in patients with sickle cell anemia and thalassemia.^5^ Autoimmune hemolytic anemia, also a cause of chronic intravascular hemolysis and associated with inflammatory bowel disease, was considered; however, our patient’s direct Coombs test was negative.^6^ A peripheral smear demonstrated polychromasia, without evidence of hemolysis. The remainder of the hemolytic evaluation including haptoglobin, indirect bilirubin, and reticulocyte count were otherwise supportive of a hemolytic process.

Evaluation for porphyria cutanea tarda was pursued at the recommendation of Hematology. Plasma porphyrins were elevated but both urine and fecal porphyrins were normal. The elevated plasma porphyrins were attributed to our patients liver disease and use of a sulfonamide drug, both of which are known to increase plasma porphyrins.

Whole genome sequencing was significant for a de novo pathogenic heterozygous c.5266C>T, p.Arg1756Ter variant in the SPTB gene, the gene that encodes a spectrum protein which is essential to cell membrane structure. Pathogenic variants of this gene have been associated with autosomal dominant hereditary spherocytosis type 2, a known cause of hemolysis. In light of the genetic findings, a subsequent review of the peripheral smear demonstrated a population of spherocytes. Baseline morphologic examinations have shown 45% of people with hereditary spherocytosis have increased spherocytes on peripheral smear.^7^ Spherocytes may represent only a small percentage of overall erythrocytes and spherocytes are one of the most common artifacts in peripheral blood smears.^8^ A specimen with prolonged storage or suboptimal staining will produce spherocytes, and was likely the reason a clinically significant population of spherocytes was not reported in the original peripheral blood smear report. Our patient’s anemia was due to hemolysis from hereditary spherocytosis. However, anemia from hereditary spherocytosis occurs from extravascular hemolysis through phagocytic degradation of erythrocytes in the spleen, and not intravascular hemolysis.^9^ Renal hemosiderosis, which was the original finding that suggested a hemolytic process, on the contrary, is typically seen in conditions of intravascular hemolysis.^5^ On review of the literature, there have been rare reports of renal hemosiderosis occurring in patients with hereditary spherocytosis.^10^

## ACKNOWLEDGMENTS

M.C. and L.C. conceptualized this case report, drafted the initial manuscript, and reviewed and revised the manuscript. S.T. reviewed and revised the manuscript. All authors approved the final manuscript as submitted and agree to be accountable for all aspects of the work.

The parents of the subject of this case report provided informed consent to publish the included information.

## References

[1] Miller SD Cuffari C Akhuemonkhan E. Anemia screening, prevalence, and treatment in pediatric inflammatory bowel disease in the United States, 2010-2014. Pediatr Gastroenterol Hepatol Nutr. 2019; 22:152–161. 30899691 10.5223/pghn.2019.22.2.152 PMC6416389

[2] Murawska N Fabisiak A Fichna J. Anemia of chronic disease and iron deficiency anemia in inflammatory bowel diseases: pathophysiology, diagnosis, and treatment. Inflamm Bowel Dis. 2016; 22:1198–1208. 26818422 10.1097/MIB.0000000000000648

[3] Goldblum JR Odze RD. Odze and Goldblum Surgical Pathology of the GI tract, Liver, Biliary Tract, and Pancreas. Philadelphia, PA: Elsevier Saunders; 2015:1525.

[4] Bassett ML Halliday JW Powell LW. Value of hepatic iron measurements in early hemochromatosis and determination of the critical iron level associated with fibrosis. Hepatology. 1986; 6:24–29. 3943787 10.1002/hep.1840060106

[5] Schein A Enriquez C Coates TD. Magnetic resonance detection of kidney iron deposition in sickle cell disease: a marker of chronic hemolysis. J Magn Reson Imaging. 2008; 28:698–704. 18777554 10.1002/jmri.21490 PMC2597353

[6] Shashaty GG Rath CE Britt EJ. Autoimmune hemolytic anemia associated with ulcerative colitis. Am J Hematol. 1977; 3:199–208. 341696 10.1002/ajh.2830030212

[7] Oliveira A Freire P Souto P. Association between the location of colon polyps at baseline and surveillance colonoscopy - a retrospective study. Rev Esp Enferm Dig. 2016; 108:563–567. 27604266 10.17235/reed.2016.4095/2015

[8] King MJ Garçon L Hoyer JD; International Council for Standardization in Haematology. ICSH guidelines for the laboratory diagnosis of nonimmune hereditary red cell membrane disorders. Int J Lab Hematol. 2015; 37:304–325. 25790109 10.1111/ijlh.12335

[9] Bolton-Maggs PH Langer JC Iolascon A; General Haematology Task Force of the British Committee for Standards in Haematology. Guidelines for the diagnosis and management of hereditary spherocytosis–2011 update. Br J Haematol. 2012; 156:37–49. 22055020 10.1111/j.1365-2141.2011.08921.x

[10] Leonardi P Ruol A. Renal hemosiderosis in the hemolytic anemias: diagnosis by means of needle biopsy. Blood. 1960; 16:1029–1038. 14415802

